# Supplementary material for: Structural journey of an insecticidal protein against western corn rootworm
Source: Nat Commun. 2023 Jul 13;14:4171. doi: 10.1038/s41467-023-39891-7 (PMC10344926; doi:10.1038/s41467-023-39891-7)
Supplement: Supplementary file 1 — Supplementary information [file 41467_2023_39891_MOESM1_ESM.pdf]

Structural journey of an insecticidal protein against western corn rootworm

Supplementary information

G. Marini[1,2], B. Poland[3], C. Leininger[3†], N. Lukoyanova[1], D. Spielbauer[3], J. Barry[3], D. Altier[3], A. Lum[3‡], E. Scolaro[3], C. Pérez Ortega[3§], N. Yalpani[3¶], G. Sandahl[3], T. Mabry[4], J. Klever[3], T. Nowatzki[3], J-Z. Zhao[3], A. Sethi[3], A. Kassa[3], V. Crane[3], A. Lu[3], M.E. Nelson[3]*, N Eswar[3]*, M. Topf[1,2]*, H.R. Saibil[1]*

*Corresponding authors. Email: mark.e.nelson@corteva.com; narayanan.eswar@corteva.com; maya.topf@cssb-hamburg.de; h.saibil@bbk.ac.uk.

This PDF file includes:

- Supplementary Figures 1 to 10
- Supplementary Tables 1 to 6
- Supplementary Results & Discussion
- Supplementary Methods
- Supplementary References ([1-37])

Other Supplementary Materials for this manuscript include:

- Supplementary Movies 1 to 3

Supplementary Figures

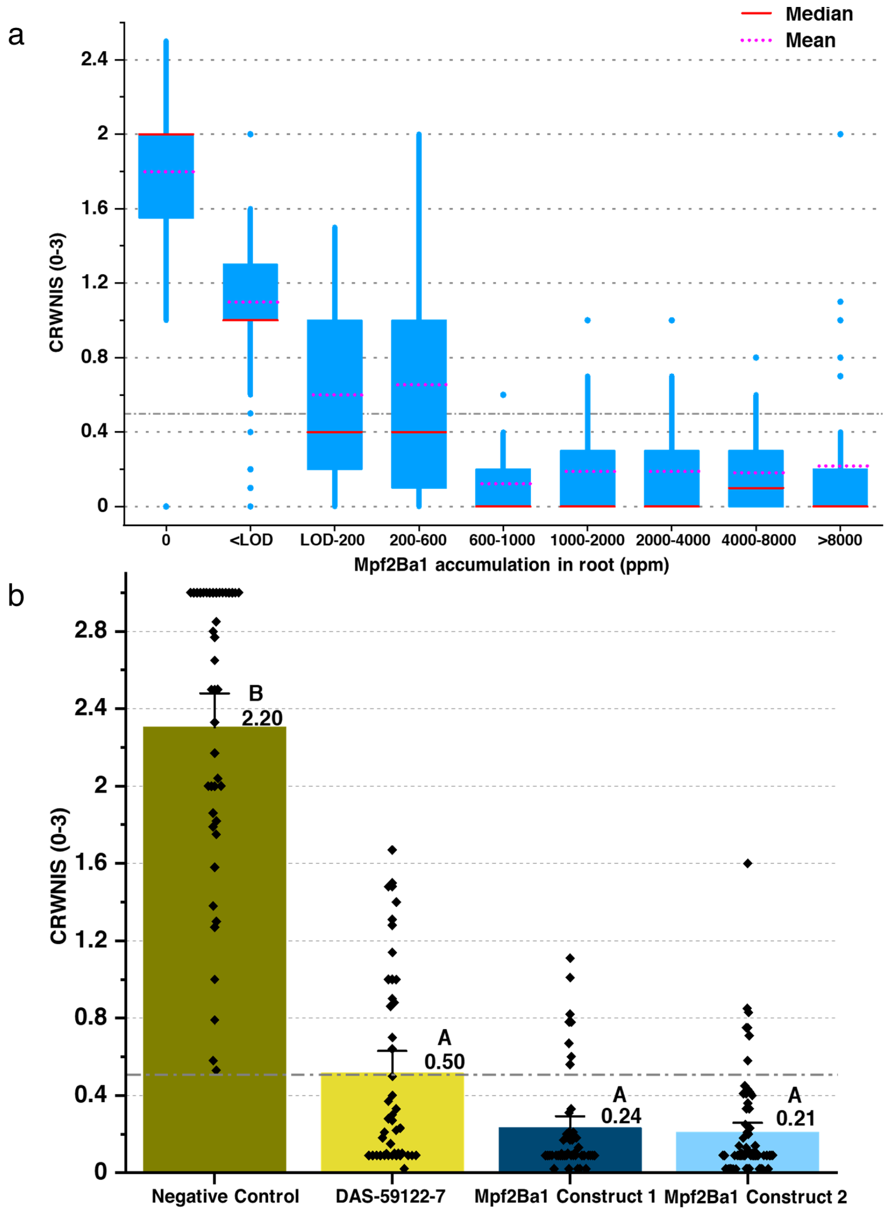

Supplementary Figure 1 | Mpf2Ba1 root expression vs. corn root worm nodal injury score (CRWNIS). a, *In planta* efficacy data expressed as the corn rootworm node injury score1 as a function of protein accumulation (mass spectrometry) in the root determined using the peptide sequence EPTPPGYTK as described previously in Hu and Owens, 2011[2]. The boxes reflect the 25-75 percentiles, and the whiskers reflect the 1.5× interquartile boundaries with values outside

these boundaries represented by solid circles. The median values are represented by solid red lines and mean values are represented by dotted magenta lines; x=0 (n=75), x=<LOD (n=58), x=LOD-200 (n=9), x=200-600 (n=11), x=600-1000 (n=13), x=1000-2000 (n=47), x=2000-4000 (n=27), x=4000-8000 (n=11), x>8000 (n=48). Plants expressing Mpf2Ba1 at 600-1000 ppm show preferred levels of rootworm control (CRWNIS < 0.5 indicated by the horizontal dashed line). Zero ppm reflects plants that were not transformed with the *mpf2ba1* gene while LOD is limit of detection by mass spectrometry. **b**, Field efficacy data comparing plants expressing Mpf2Ba1 (two different constructs) to DAS-59122, the commercial event that expresses Gpp34Ab1/Tpp35Ab1, and untreated control hybrid plants (Negative Control). The bars reflect the mean for data collected for each treatment from four different locations in 2016 and the error bars reflect SEM while the solid black diamonds represent scores for each plant (n=42, 45, 47, and 69 for Negative Control, DAS-59122-7, Construct 1, and Construct 2, respectively). The mean values from the linear mixed model are provided along with letters that indicate whether differences between treatments are significant as described in Methods. Source data are provided within the Source Data file.

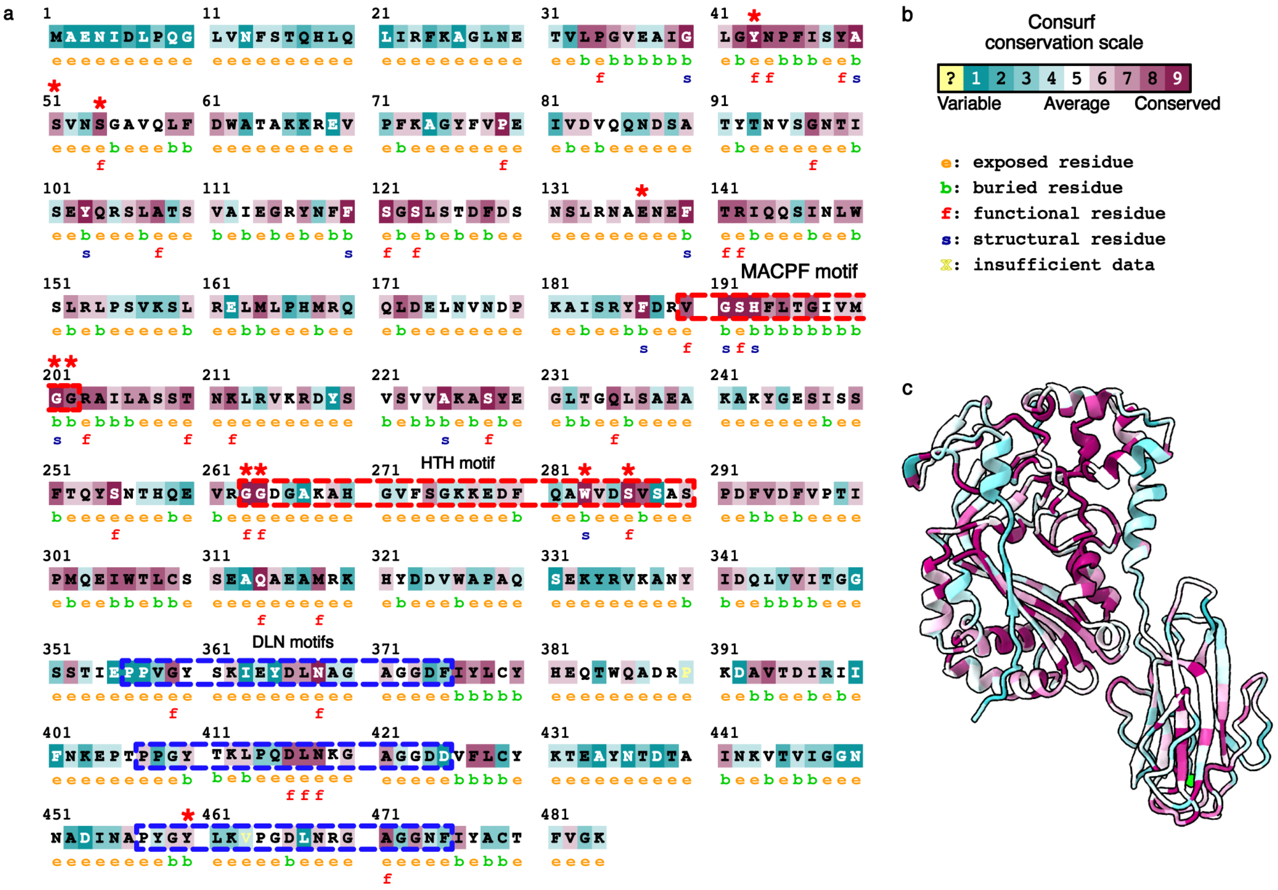

Supplementary Figure 2 | Mpf2Ba1 sequence conservation and residue evolution among bacteria. a, Each sequence position of Mpf2Ba1 is colored according to conservation scores obtained using ConSurf[3], to show residue conservation and evolutionary rate. Red stars highlight key conserved residues: glycines 201-202, in the MACPF central sheet, glycines 263-264 and Trp283[4] and Ser286 in the HTH motif, and the conserved H-bond between Glu137 and Tyr460. Red dashed boxes frame the sequences of MACPF signature motif Y/W-G-T/S-H-F/Y-X_6-GG[4,5] which in Mpf2Ba1 diverges slightly from the consensus with a non-conserved change from Tyr/Trp to Val ($_{190}$V-G-S-H-$X_{6(FLTGIVM)}$-GG_{202}), and the sequence of the HTH motif (see also Supplementary Fig. 3a, red dashed boxes); blue dashed boxes frame the sequences of Mpf2Ba1 C-terminal repeated MABP motif PxG(Y/F)(T/S)xIxxDLNxxxxGx(Y/F). **b**, Consurf conservation

scale and legend[3]. **c**, The X-ray crystal structure of Mpf2Ba1 has been rendered by color coding attribute using ChimeraX v1.4[6] to reflect the evolutionary conservation.

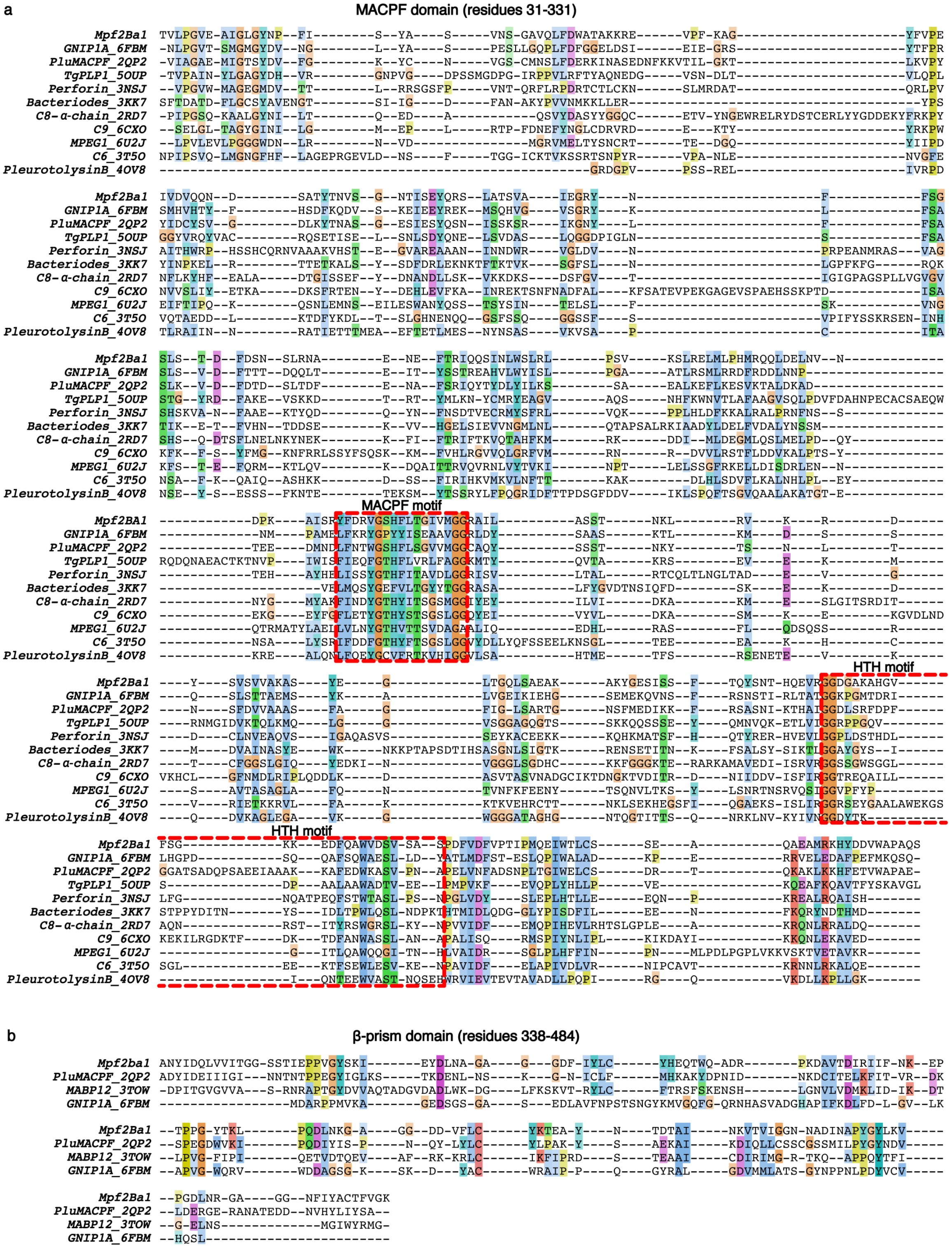

Supplementary Figure 3 | Sequence alignment and residue conservation between MACPFs. **a**, Mpf2Ba1 MACPF domain sequence aligned to the closest structural homologues using HHPred[7]

and visualized in Jalview[8]: Mpf3Aa1 (GNIP1Aa, PDB ID: 6FBM)[9], Mpf1Aa1 (Plu-MACPF from *Photorhabdus luminescens*, PDB ID: 2QP2)[10], *Toxoplasma gondii* perforin-like protein 1 (PDB ID: 5OUP)[11], human perforin (PDB ID: 3NSJ)[12], MACPF *Bacteroides thetaiotaomicron* (PDB ID: 3KK7)[4], human C8 protein (PDB ID: 2RD7)[13], mouse complement component-9 (PDB ID: 6CXO)[14], MPEG1/Perforin-2 (PDB IDs: 6U23 and 6SB3)[15,16], human C6 protein (PDB ID: 3T5O)[17], and pleurotolysin B (PDB ID: 4OV8)[18]. Red stars highlight key conserved residues: glycines 201-202, in the MACPF central sheet, glycines 263-264 and Trp283[4] in the HTH motif, and the conserved H-bond between Glu137 and Tyr460. Red dashed boxes frame the sequence of MACPF signature motif Y/W-G-T/S-H-F/Y-X_6-GG[10,19], which in Mpf2Ba1 has a non-conserved change from Tyr/Trp to Val ($_{190}$V-G-S-H-$X_{6(FLTGIVM)}$-GG_{202}), and the sequence of the HTH motif. **b**, Mpf2Ba1 β-prism domain sequence aligned to the closest structural homologues MACPF/CDCs: Mpf1Aa1 (PDB ID: 2QP2)[10], Mpf3Aa1 (PDB ID: 6FBM)[9], and the multivesicular body subunit 12 MABP domain of Human ESCRT-I complex (PDB ID: 3TOW)[20].

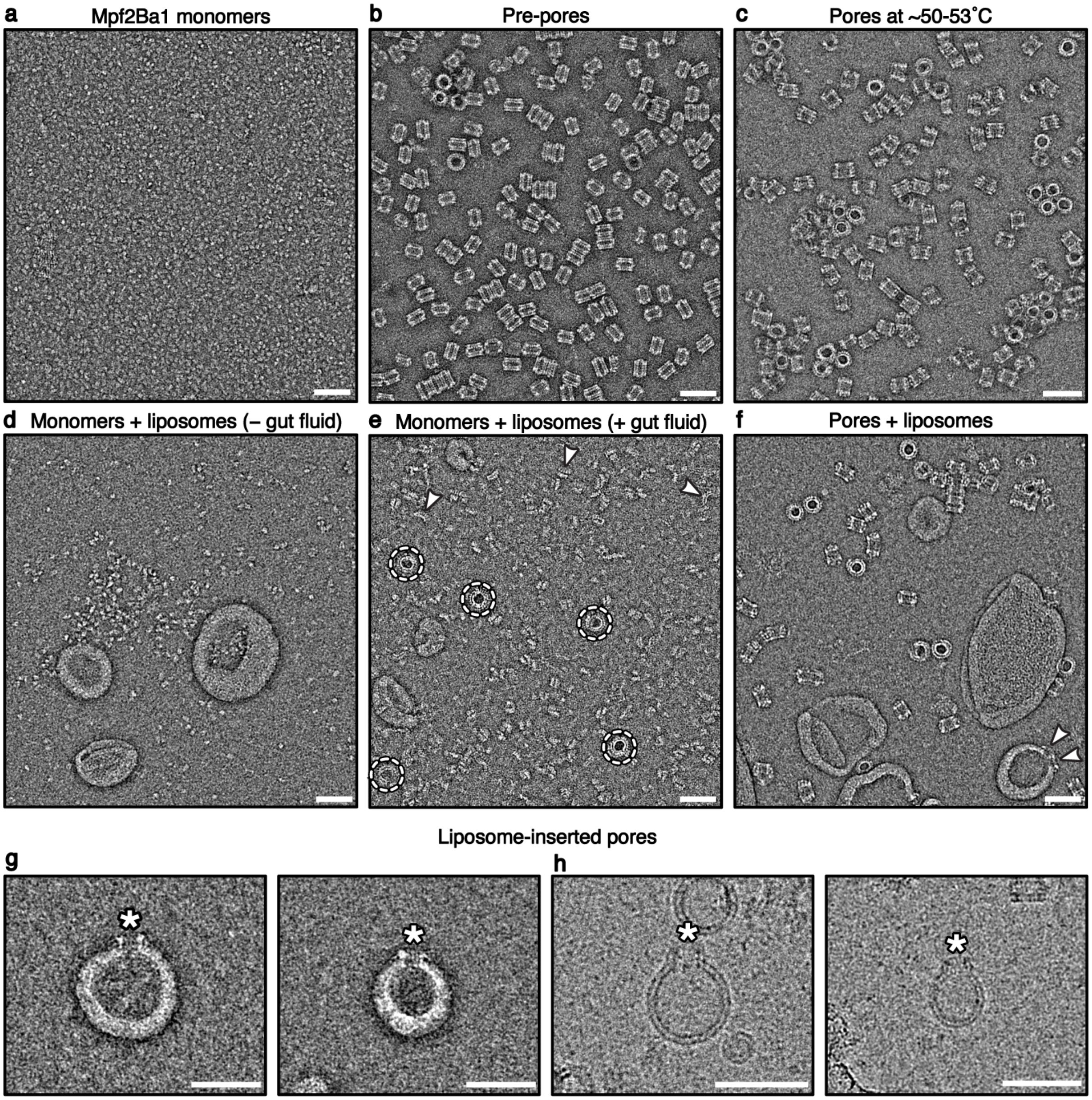

Supplementary Figure 4 | Negative stain EM to assess pore sample preparation. The micrographs show Mpf2Ba1 **a**, soluble monomers alone; **b**, pre-pores oligomerized from monomers incubated in WCR gut fluid; **c**, pores converted from pre-pores using the heating method without liposomes/lipids; **d**, monomers incubated in POPC and 4% cholesterol liposomes without gut fluid, showing no oligomerization; **e**, monomers activated by gut fluid and incubated in liposomes, showing weak oligomerization into pores (white dashed circles) and small arc-like

assemblies, (white arrowheads); **f**, pores converted from pre-pores after incubation in liposomes, (white arrowheads indicate liposome-inserted pores); n≥5. **g** and **h**, Magnified views of liposome-inserted Mpf2Ba1 pores (white stars) imaged by negative stain in (**g**) and by cryo-EM in (**h**). Scale bars: 50 nm; n≥5.

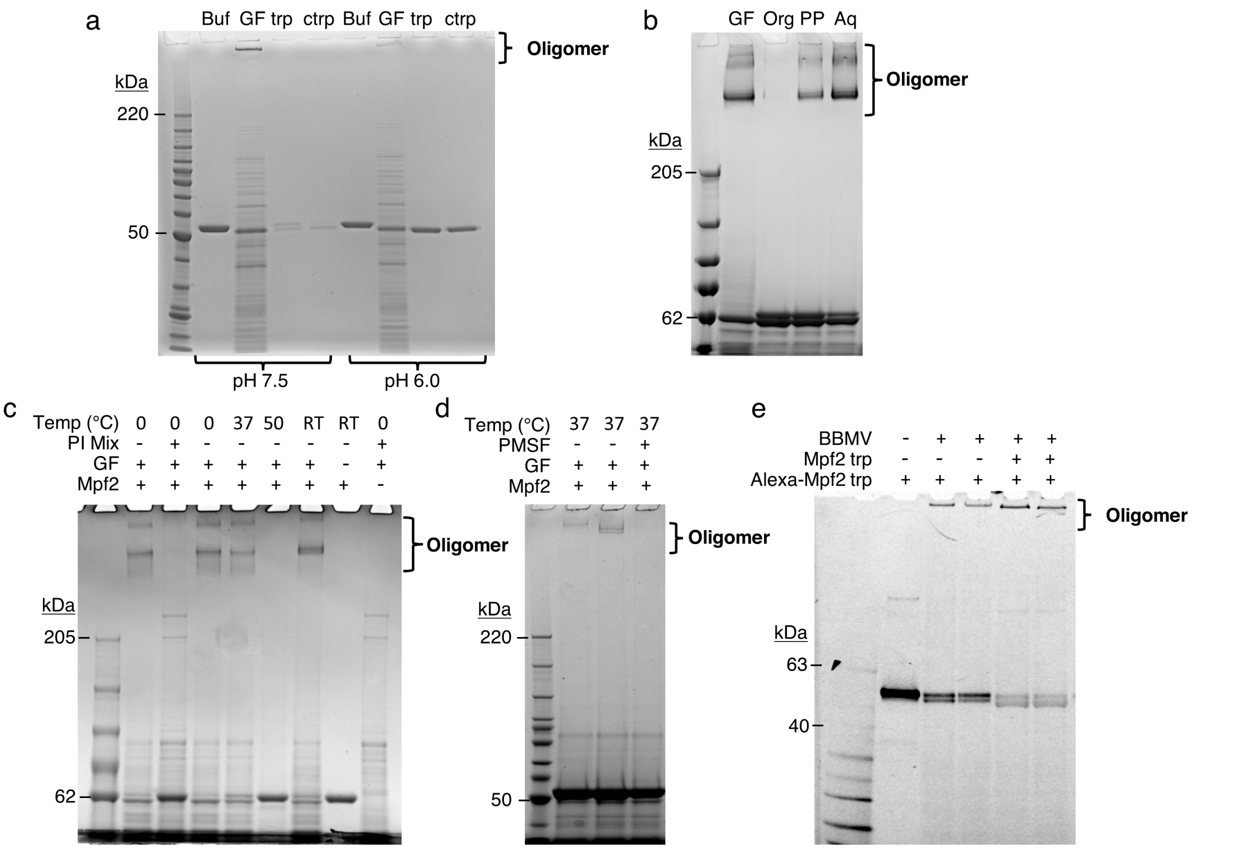

Supplementary Figure 5 | Characterizing oligomer formation by Mpf2Ba1 when exposed to WCR gut fluid. a, Image of Coomassie stained denaturing polyacrylamide gel showing full-length Mpf2Ba1 treated with buffer only (Buf), gut fluid (GF), immobilized trypsin agarose (trp) or immobilized chymotrypsin agarose (ctrp) in either phosphate buffer, pH 7.5 or Bis-Tris buffer, pH 6.0. Both trypsin and chymotrypsin treatments produced a truncated form of Mpf2Ba1 similar in size to that produced by gut fluid treatment, but the oligomer was only present in the gut fluid treated samples. Similar results for GF- and trp-treated Mpf2Ba1 were observed in numerous experiments, while ctrp effects were tested in one other experiment. **b**, Image of Coomassie stained denaturing polyacrylamide gel showing full-length Mpf2Ba1 treated with gut fluid (GF), or the different phase separations following gut fluid subjected to chloroform:methanol:water (2:1:1) extraction. "Org" represents treatment with the buffer reconstituted organic phase of the extraction

following dry-down under a stream of argon, “PP” represents the protein enriched interphase of the extraction resuspended in buffer, and “AQ” represents the aqueous phase. Similar results were observed in four other experiments. **c**, Image of Coomassie stained denaturing polyacrylamide gel showing full-length Mpf2Ba1 (Mpf2) treated with gut fluid (GF) at different temperatures (0, 37, or 50°C, or room temperature- RT) and in the presence of cOmplete^{TM} protease inhibitor cocktail (PI Mix) at 0°C. Gut fluid-induced oligomerization was prevented by the protease inhibitors or when protease activity was inactivated by heating to 50°C. Similar results for temperature effects were observed in three other experiments, while similar effects of PI Mix were observed in four other experiments. **d**, Image of Coomassie stained denaturing polyacrylamide gel showing full-length Mpf2Ba1 (Mpf2) treated with gut fluid (GF) in absence and presence of serine protease inhibitor phenylmethylsulfonyl fluoride (PMSF; 5 mM). Oligomerization was prevented by the addition of PMSF to GF. Similar results with PMSF-treated GF were observed in two other experiments. **e**, Image of in-gel fluorescence of a denaturing polyacrylamide gel showing Alexa-labeled Mpf2Ba1 (Alexa-Mpf2) (20 nM) after processing by immobilized trypsin agarose by itself (10% of 20 nM) or after binding reactions with WCR BBMVs (20 mg) in the absence or presence of unlabeled Mpf2Ba1 (10 mM) with the final pellets solubilized in sample buffer prior to electrophoresis. The appearance of the high molecular weight oligomer occurs in the presence of BBMVs. Similar results for oligomer formation by Mpf2Ba1trp were observed in four other experiments. The first lane of all gels contained molecular weight size standards.

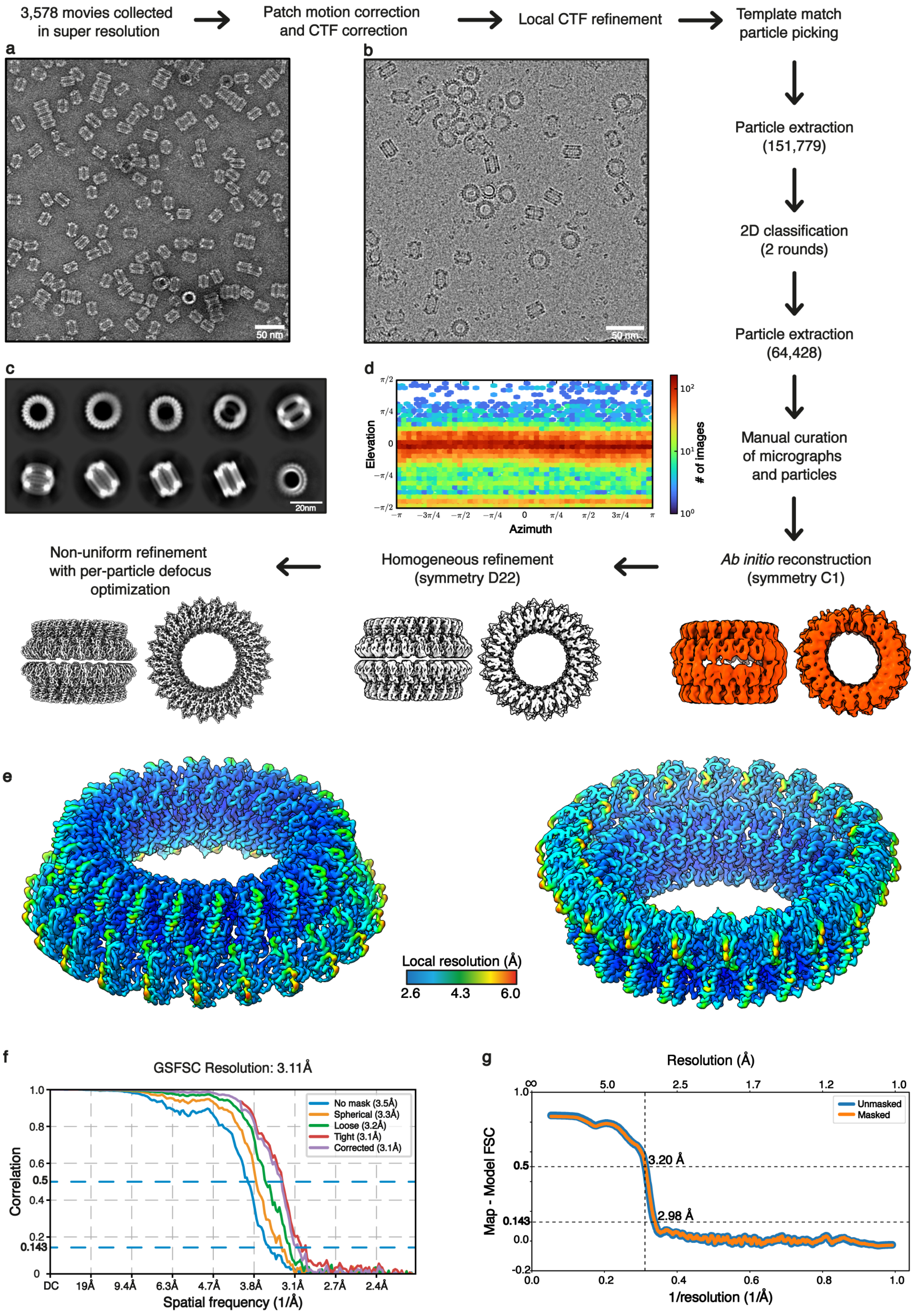
3,578 movies collected in super resolution
Patch motion correction and CTF correction
Local CTF refinement
Template match particle picking
a
b
50 nm
50 nm
Particle extraction (151,779)
2D classification (2 rounds)
Particle extraction (64,428)
c
d
20nm
Elevation
Azimuth
# of images
Manual curation of micrographs and particles
Ab initio reconstruction (symmetry C1)
Homogeneous refinement (symmetry D22)
Non-uniform refinement with per-particle defocus optimization
e
Local resolution (Å)
2.6
4.3
6.0
f
GSFSC Resolution: 3.11Å
No mask (3.5Å)
Spherical (3.3Å)
Loose (3.2Å)
Tight (3.1Å)
Corrected (3.1Å)
Correlation
Spatial frequency (1/Å)
DC
19Å
9.4Å
6.3Å
4.7Å
3.8Å
3.1Å
2.7Å
2.4Å
g
Resolution (Å)
Unmasked
Masked
Map - Model FSC
3.20 Å
2.98 Å
1/resolution (1/Å)

Supplementary Figure 6 | Single particle analysis and processing pipeline for the Mpf2Ba1 pre-pore dataset. **a** and **b**, Sample micrographs of pre-pores obtained upon incubation in WCR gut fluid (see Methods) and imaged by negative stain EM in (**a**) and by cryo-EM in (**b**); n≥5. **c**, 2D class averages of pre-pores selected for refinement after the last round of classification. **d**, Plot of the angular distribution of particles contributing to the structure of the pre-pore. **e**, Local resolution estimation of the pre-pore map. **f**, FSC curve of the pore showing the final average resolution for the map (3.1 Å at gold standard FSC = 0.143). **g**, Plot of the map-to-model FSC with and without mask showing very similar FSC curves and resolutions. Details are described in Methods and Supplementary Table 5.

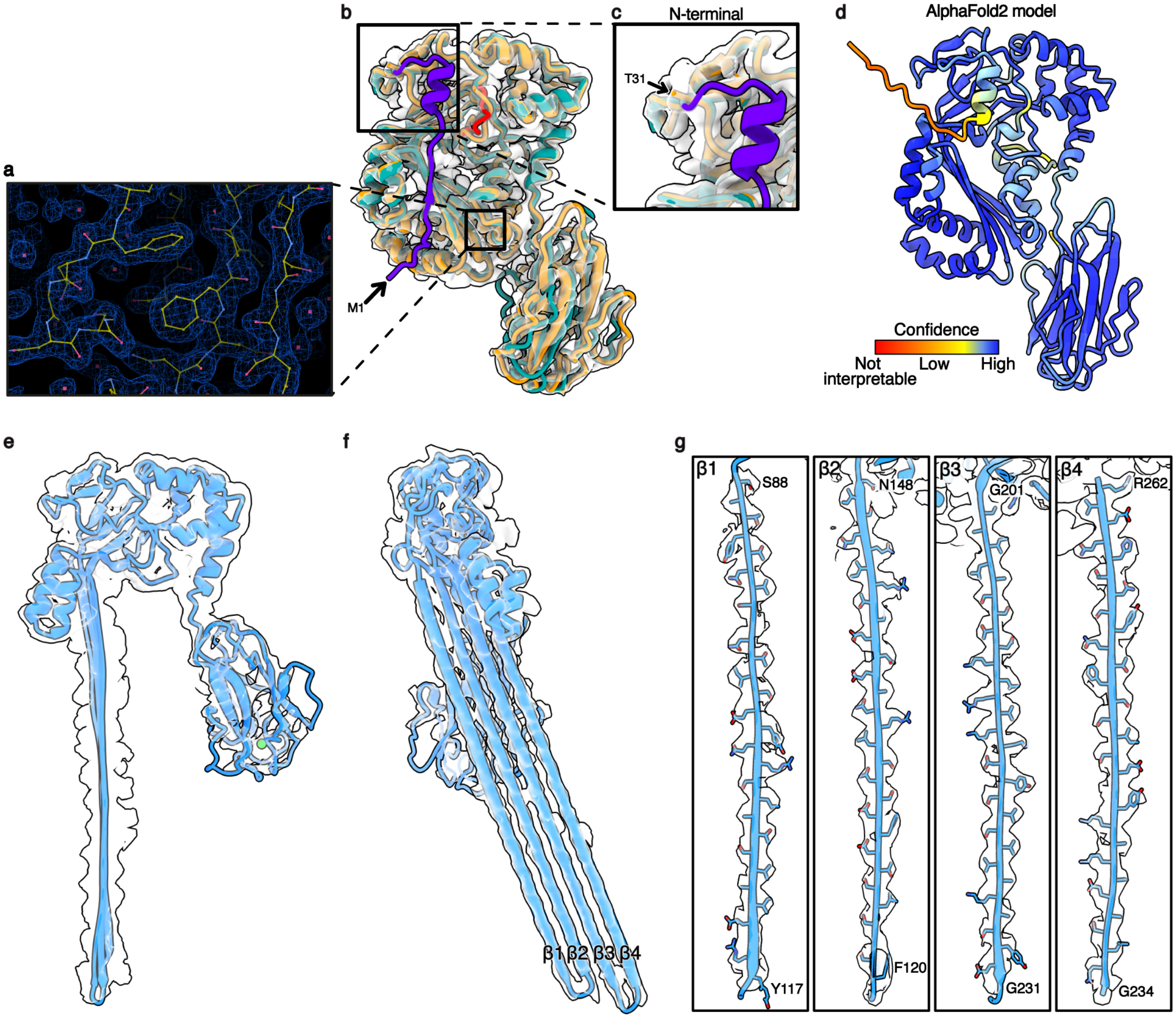

Supplementary Figure 7 | Mpf2Ba1 putative N-terminal cleavage site, AlphaFold2 prediction and comparison. a, Image of representative 2Fo-Fc electron density for Mpf2Ba1-1167 crystal structure contoured to 1.2 RMSD, showing central residues F128 and F140. **b**, Superimposition of Mpf2Ba1-1167 soluble monomer (in cyan) onto pre-pore model (in orange) fitted in the map of a single subunit shows that the first 30 residues of the soluble monomer, from Met1 (black arrow), are absent in the pre-pore map density (in purple). **c**, Magnified inset showing the density of the pre-pore map starting at N-terminal Thr31 (arrow pointing at Thr31). **d**, AlphaFold2[21] predicts the N-terminal α-helix but not the short β-strand Val12-Ser15, where those and other residues are

predicted with low confidence and not in contact with neighbouring loops/domains. The predicted model has an all-atom RMSD of 2.1 Å and 1.8 Å, for soluble monomer and pre-pore respectively. **e**, Pore model fit into the cryo-EM map for a single subunit. **f**, Individual map and models of β-hairpins 1 to 4 showing the amino acid side chains matching the map densities.

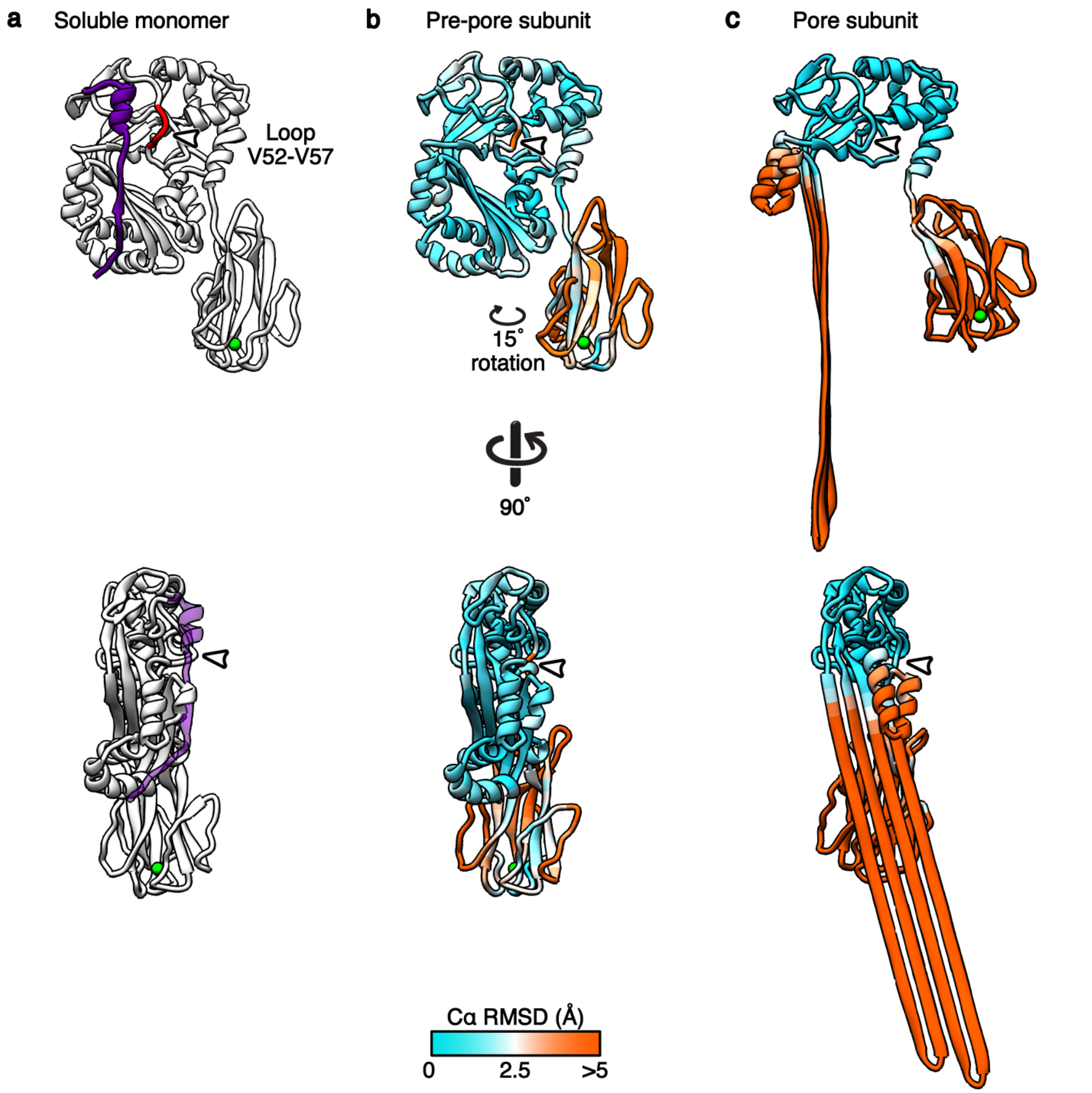

Supplementary Figure 8 | Cα RMSD plots of the displacements between Mpf2Ba1 conformations. **a**, 30 N-terminal residues in the Mpf2Ba1-1167 soluble monomer (in purple) are not present in the pre-pore and pore models where there is no cryo-EM density to include them; the loop Val52-Val57 is highlighted in red (white arrowhead). **b**, In the pre-pore the MACPF domain is mostly unchanged (1.2 Å average Cα RMSD for residues 31 to 337, in cyan), except

from the loop Val52-Val57 (4.0 Å average Cα RMSD; white arrowheads), and the C-terminal MABP domain that rotates ~15° clockwise relative to the monomeric structure (4.2 Å average Cα RMSD for residues 338 to 484). **c**, In the pore structure, the upper part of the MACPF domain remains unchanged (1.4 Å Cα RMSD) while TMHs undergo a big conformational change into β-hairpins, and C-terminal domain and HTH motif move ~12 Å and ~8 Å respectively from their center of mass compared to the pre-pore conformation. The Cα RMSD distances were calculated in Chimera v1.15[22] between soluble monomer and pre-pore, reported in (**b**) and between pre-pore and pore, reported in (**c**).

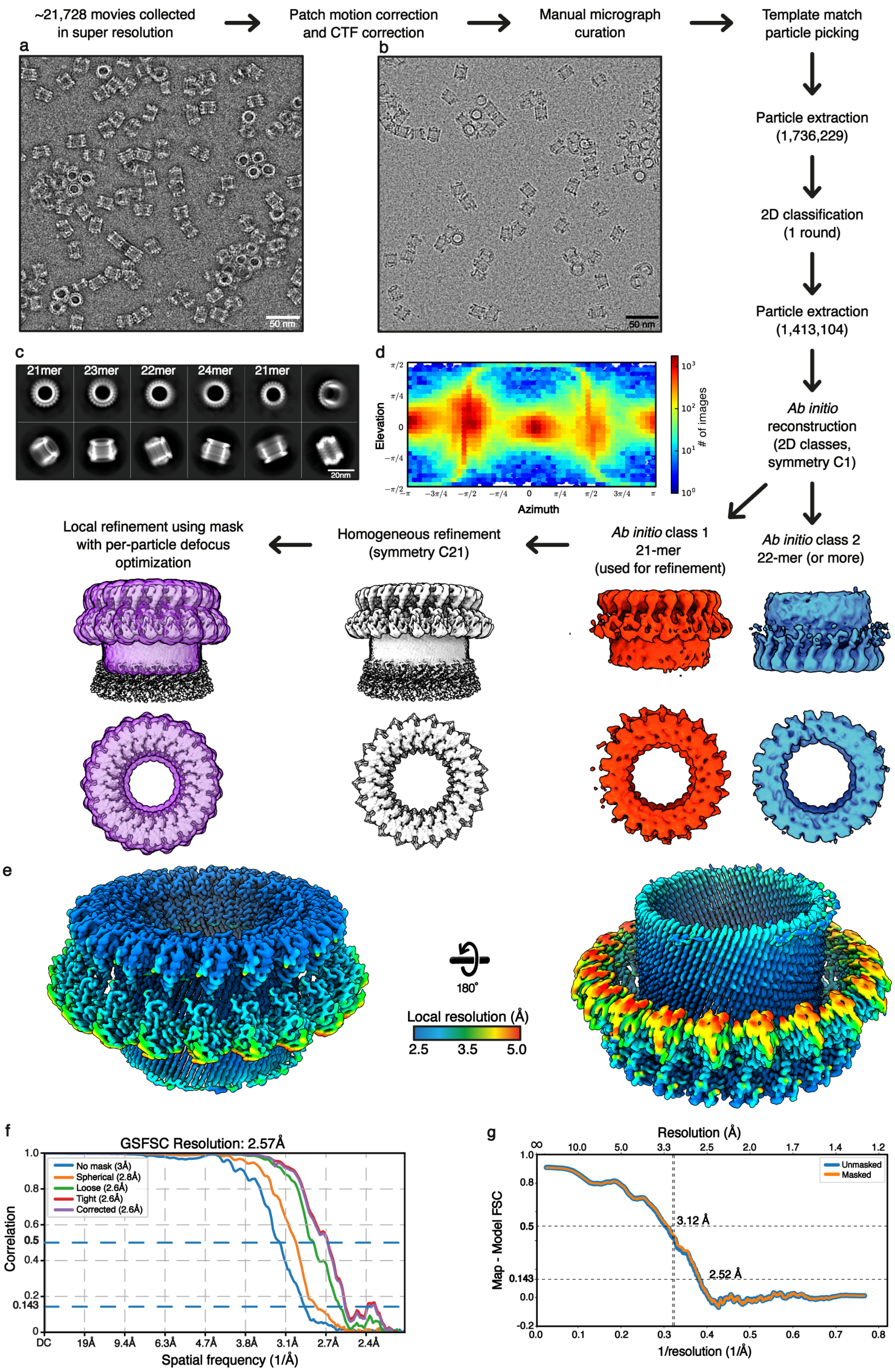
~21,728 movies collected in super resolution
Patch motion correction and CTF correction
Manual micrograph curation
Template match particle picking
Particle extraction (1,736,229)
2D classification (1 round)
Particle extraction (1,413,104)
Ab initio reconstruction (2D classes, symmetry C1)
Ab initio class 1 21-mer (used for refinement)
Ab initio class 2 22-mer (or more)
Homogeneous refinement (symmetry C21)
Local refinement using mask with per-particle defocus optimization
a
b
50 nm
c
21mer
23mer
22mer
24mer
21mer
20nm
d
Elevation
Azimuth
# of images
e
180˚
Local resolution (Å)
2.5
3.5
5.0
f
GSFSC Resolution: 2.57Å
No mask (3Å)
Spherical (2.8Å)
Loose (2.6Å)
Tight (2.6Å)
Corrected (2.6Å)
Correlation
Spatial frequency (1/Å)
g
Resolution (Å)
Map - Model FSC
1/resolution (1/Å)
Unmasked
Masked
3.12 Å
2.52 Å

Supplementary Figure 9 | Single particle analysis and processing pipeline for the Mpf2Ba1 pore dataset. **a** and **b**, Sample micrographs of pores obtained upon incubation at ~50-53˚C (see Methods), imaged by negative stain EM in (**a**) and by cryo-EM in (**b**); n≥3. **c**, 2D class averages of pores obtained at the last round of classification showed a distribution of oligomer stoichiometries, ranging from the most populated 22-fold symmetry (52% of end view particles) to 21-fold (30%), 23-fold (11%) and 24-fold (7%), demonstrating heterogeneity of oligomerization. The mask created for the local refinement to enhance the density of the 21-mer pore is shown in violet. **d**, Plot of the angular distribution of particles contributing to the structure of the pore. **e**, Local resolution estimation of the pore map. **f**, FSC curve of the pore showing the final average resolution for the map (2.6 Å at FSC = 0.143). **g**, Plot of the map-to-model FSC with and without mask showing very similar FSC curves and resolutions. Details are described in Methods and Supplementary Table 5.

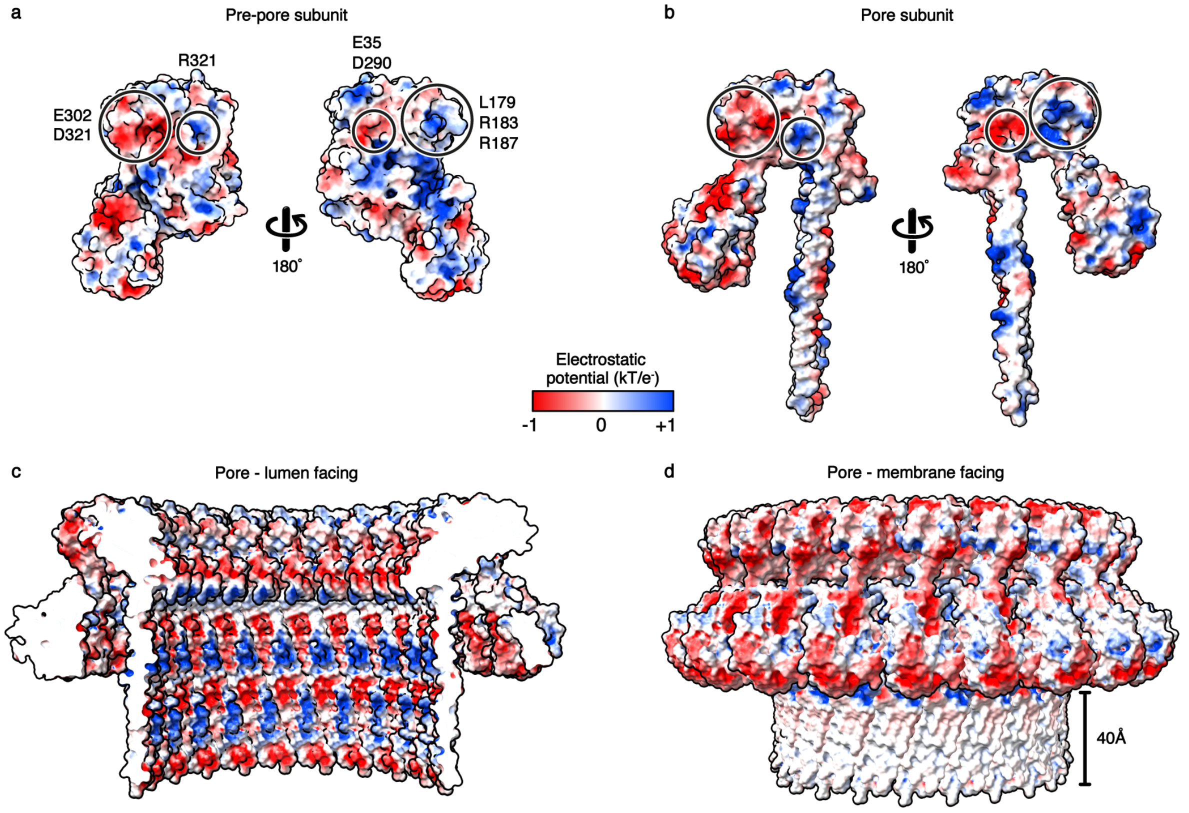

Supplementary Figure 10 | Analysis of the electrostatic potential maps of Mpf2Ba1 pre-pore and pore. a and **b**, MACPF domains of neighbouring pre-pore and pore subunits show opposite charges in complementary sites of interaction: on the left, the negative pocket of Glu302, Asp321 and the positive charge of Arg151 match exactly with the positive pocket of Lys179, Arg183 and Arg187, and the negative pocket of Glu35 and Asp290, on the right (black circles). **c**, The distribution of charges for the entire pore structure shows the highly polar lumen-facing part, including charged residues such as Glu, Asp, Lys and up to 9 Ser per subunit, while in (**d**) the membrane-facing region has a charge-neutral belt, ~40 Å in height, enriched in Phe, Tyr, Val and Ile hydrophobic residues.

Supplementary Tables

Supplementary Table 1.

Conservative mutations of Mpf2Ba1-1167 relative to the wild-type protein.

	Amino acid position								
	**81**	**92**	**100**	**322**	**334**	**340**	**347**	**435**	**454**
**Mpf2Ba1 WT**	I	Y	I	Y	Y	Y	I	Y	I
**Mpf2Ba1-1167**	L	F	L	F	F	F	L	F	L

Supplementary Table 2.

Mpf2Ba1 bioactivity in artificial diet bioassays (IC_{50} and LC_{50}) against *Diabrotica virgifera* (WCR), *Diabrotica barberi* (NCR) and *Diabrotica speciosa.*

*Mortality was 35% at 400 ppm dose.

	**LC/IC$_{50}$**	**WCR (ppm)**	**NCR (ppm)**	***Diabrotica speciosa* (ppm)**
**Mpf2Ba1 WT**	LC_{50}	16.3	35.6	>400*
	IC_{50}	7.4	13	320

Supplementary Table 3.

Bioactivity of Mpf2Ba1 and Mpf2Ba1-1167 against susceptible and Readlyn populations of WCR. Artificial diet bioassay data indicate that Mpf2Ba1 and Mpf2Ba1-1167 are not cross-resistant to the Cry3A and Gpp34Ab1/Tpp35Ab1 proteins. Resistance ratio (RR) defined as the LC_{50} value determined for resistant WCR divided by the LC_{50} value determined for susceptible WCR. ND: could not determine.

**Protein**	**WCR Population**	**LC_{50}, µg/ml**	**95% Confidence Interval**	**Slope ± Standard Error**	**n**	**Resistance Ratio (RR)**
**Gpp34Ab1/Tpp35Ab1**	Susceptible	5.90	3.06 - 8.60	3.27 ± 0.910	144	8
	Readlyn	45.7	23.6 - 85.3	1.08 ± 0.207	144	
**mCry3A**	Susceptible	6.97	1.61 - 13.7	1.90 ± 0.600	140	43
	Readlyn	302	87.1 - 6680	0.644 ± 0.230	212	
**Mpf2Ba1 WT**	Susceptible	10.6	8.22 - 13.6	2.34 ± 0.277	287	0.7
	Readlyn	7.46	5.62 - 9.72	2.32 ± 0.290	288	
**Mpf2Ba1-1167**	Susceptible	14.3	ND	ND	144	0.8
	Readlyn	11.4	6.80 - 16.9	2.07 ± 0.397	144	

Supplementary Table 4.

Data collection and refinement statistics of Mpf2Ba1-1167 (molecular replacement).

	Mpf2Ba1-1167 monomer (PDB ID: 8B6U)
**Data collection**	
Space group	P 41 21 2
Cell dimensions	
a, *b*, *c* (Å)	127.61, 127.61, 116.12
α, β, γ (°)	90, 90, 90
Resolution (Å)	2.13 (high res. shell)
R_{sym} or R_{merge}	0.104 (high res. shell)
$I / \sigma I$	10.8 (high res. shell)
Completeness (%)	99.6 (high res. shell)
Redundancy	4.6 (high res. shell)
**Refinement**	
Resolution (Å)	2.134
No. reflections	53424
R_{work} / R_{free}	0.2094/0.2414
No. atoms	
Protein	3730
Ligand/ion	1
Water	204
B-factors	
Protein	33.35
Ligand/ion	22.31
Water	33.53
R.m.s. deviations	
Bond lengths (Å)	0.0120
Bond angles (°)	1.8337

Supplementary Table 5.

Cryo-EM data collection, refinement, and validation statistics of Mpf2Ba1 pre-pore and pore.

	Mpf2Ba1 pre-pore (EMDB-15882; PDB ID: 8B6V)	Mpf2Ba1 pore (EMDB-15883; PDB ID: 8B6W)
**Data collection and processing**		
Magnification	×130k	×81k
Voltage (kV)	300	300
Electron exposure (e–/Å^2)	39.56	50.65
Defocus range (μm)	1.5-3.0	1.5-3.3
Pixel size (Å)	1.05	1.067
Symmetry imposed	D22	C21
Initial particle images (no.)	151,779	1,736,229
Final particle images (no.)	64,248	1,413,104
Map resolution (Å)	3.1	2.6
FSC threshold	0.143	0.143
Map resolution range (Å)	2.5-4.5	2.5-6.0
**Refinement**		
Initial model used (PDB code)	8B6U – Mpf2Ba1 monomer	8B6V – Mpf2Ba1 pre-pore
Model resolution (Å)	2.98	2.52
FSC threshold	0.143	0.143
Model resolution range (Å)	2.5-4.5	2.5-6.0
Map sharpening *B* factor (Å^2)	-78.5	-62.25
Model composition		
Non-hydrogen atoms	3525	3525
Protein residues	454	454
Ligands	Mg^{2+}	Mg^{2+}
B factors (Å^2)		
Protein	42.00/115.78/72.28	17.99/204.35/94.51
Ligand	100.27/100.27/100.27	196.79/196.79/196.79
R.m.s. deviations		
Bond lengths (Å)	0.003	0.004
Bond angles (°)	0.637	0.718
**Validation**		
MolProbity score	1.67	1.54
Clashscore	6.63	4.33
Poor rotamers (%)	/	/
Ramachandran plot		
Favored (%)	95.58	95.35
Allowed (%)	4.20	4.42
Disallowed (%)	0.22	0.22

Supplementary Table 6.

a, Final validation scores for the pre-pore and pore models calculated using different refinement and validation programs. **b**, SMOCf scores[23,24] are shown per residue in the plots. **c**, Backbone, side chains and residues Q-scores from MapQ[25] are calculated with the default width value of σ = 0.6 and showed averaged over 3 residues in the plots.

a

Validation scores	Pre-pore	Pore
Q-score (σ = 0.6)	0.64	0.59
Average SMOCf score	0.85	0.88
3D-Strudel score (2.8-3.0 Å motif library)	0.946	0.895
MolProbity score	1.67	1.54

b

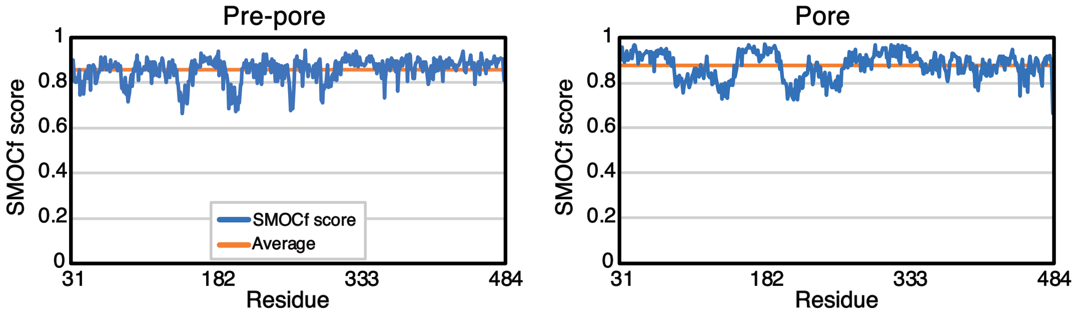

c

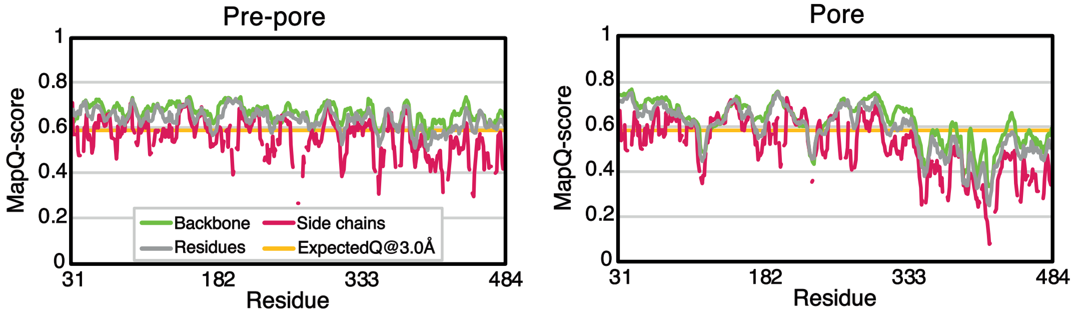

Supplementary Results & Discussion

Field efficacy

For evaluation of Mpf2Ba1-expressing plants under field conditions, second-generation (T2) non-segregating hybrid maize seeds, derived from two constructs transformed to contain Mpf2Ba1 (two events from Construct 1 and three events from Construct 2) were evaluated at 4 locations across the U.S. corn belt in 2016 (Johnston, IA; Mankato, MN; Brookings, SD, and Mansfield, IL).

The field trials were conducted on land that contained late planted conventional corn in the previous season to attract corn rootworm beetles for egg laying and to enhance natural infestations. In addition, plots at all locations were manually infested with 750 to 1,500 WCR eggs per plant (depending on location) between growth stages V2 and V4. Based on mean ± SD node-injury scores obtained from the negative control plants, corn rootworm larval feeding pressure was classified as high at Johnston, Mankato, and Brookings (2.9±0.23, 2.7±0.33 and 1.8±0.68, respectively); and moderate at Mansfield (1.5±0.45).

General observations from all locations indicated that the predominant corn rootworm species was the WCR. Combining the data across all four testing locations, plants expressing the Mpf2Ba1 protein provided very good root protection from feeding injury by corn rootworm larvae (Supplementary Fig. 1b). Details on statistics for the fixed and random effects are summarized in the Material and Methods section. Mean node-injury scores between the 2 Mpf2Ba1 constructs were numerically lower, but not significantly different from the root protection provided by the commercial hybrid corn line DAS-59122-7 across the four locations ($P < 0.05$; Supplementary Fig. 1b). Both Mpf2Ba1 constructs provided good protection compared with the negative control ($P < 0.05$, Supplementary Table 1). As reference, a node-injury score of 1.0 under field conditions has been estimated to cause a 15 to 18% reduction in corn grain yield[26,27].

Activity against resistant WCR

Using an artificial diet, we performed concentration-response bioassays and computed the LC_{50} values for Mpf2Ba1 against the susceptible rootworm population and the "Readlyn" population (Supplementary Table 3). The Readlyn population is much less sensitive to both Gpp34Ab1/Tpp35Ab1 and mCry3A with resistance ratios (LC_{50} against Readlyn population/LC_{50} against susceptible population) of 8- and 43-fold, respectively. Mpf2Ba1 or Mpf2Ba1-1167 shows a resistance ratio of <1-fold, indicating that there is no cross-resistance to mCry3Aa or Gpp34Ab1/Tpp35Ab1. These results show that the mechanism by which Mpf2Ba1 elicits mortality is different compared to either mCry3Aa or Gpp34Ab1/Tpp35Ab1.

Oligomerization of Mpf2Ba1 upon N-terminal cleavage

As stated in the main text, proteolytic activation of Mpf2Ba1 is necessary, but not sufficient to induce oligomerization. Oligomerization was first observed using denaturing polyacrylamide gel electrophoresis after incubation of the protein with gut fluid extracted from WCR larvae (Supplementary Fig. 5). The oligomerization could be prevented by treating the gut fluid with heat, a protease inhibitor cocktail (cOmplete™ EDTA-free, Roche Lifescience), or with PMSF alone (Supplementary Fig. 5). However, it was not clear whether the N-terminal truncation *per se* was triggering oligomerization or not. Therefore, Mpf2Ba1 was treated with trypsin and chymotrypsin to see if these proteases truncated the protein and whether oligomerization was observed. Both proteases truncated Mpf2Ba1 to a fragment that was very similar in size to the one observed with gut fluid processing, but neither caused oligomer formation to occur (Supplementary Fig. 5). Trypsin was then used to activate Mpf2Ba1 and the resulting peptide truncated at Ala26 was exposed to gut fluid where oligomerization was observed. Furthermore, gut fluid treated with protease inhibitors could drive oligomer formation of truncated Mpf2Ba1 indicating that additional

proteolysis was not needed for oligomerization to occur. These observations indicated that an additional factor must be present in gut fluid that interacts with activated Mpf2Ba1 and triggers oligomerization. Finally, incubation of trypsin-truncated Mpf2Ba1 with BBMVs (Supplementary Fig. 5) or with southern corn rootworm cells (Du182A) also resulted in oligomer/pore formation (Fig. 1b and c), indicating that binding to a membrane receptor triggers oligomerization.

Supplementary Methods

Identification, isolation, and purification of Mpf2Ba1

The insecticidal protein Mpf2Ba1 was identified through activity-based screening and protein purification as described in Schellenberger et al., 2016 and Wei et al., 2018[28,29]. Briefly, insecticidal activity against western corn rootworm (WCR) was measured with a cell lysate of bacterial strain JH34071-1 that was grown in Tryptic Soy broth (TSB, peptone from casein 15 g/L; peptone from soymeal 5.0 g/L; NaCl 5.0 g/L) and cultured 1 day at 28° C with shaking at 200 rpm. Bioassays with WCR were conducted by mixing the bacterial cell lysate with *Diabrotica* diet (Frontier Agricultural Sciences, Newark, DE, U.S.A.) in a 96 well format. WCR neonates were placed into each well of a 96 well plate. The assay was run for four days at 25° C and then was scored for insect mortality and stunting of insect growth. The scores were noted as dead "3", severely stunted "2" (little or no growth but alive), stunted "1" (growth to second instar but not equivalent to controls) or normal growth "0". Samples demonstrating mortality or severe stunting were studied further. Once the activity was established, the equivalent sample was subjected to heat and Pronase treatments and resubmitted for insect bioassays to confirm that the insecticidal activity was due to a proteinaceous component.

To identify the bacterial strain where activity was observed, genomic DNA of isolated strain JH34071-1 was prepared according to a library construction protocol developed by Illumina and sequenced using the Illumina® Genome Analyzer IIx (Illumina Inc., San Diego, CA, U.S.A.). The nucleic acid contig sequences were assembled and open reading frames were generated. The 16S ribosomal DNA sequence of strain JH34071-1 was BLAST™ searched against the NCBI database[30], identifying strain JH34071-1 as a *Pseudomonas monteilii* strain. To isolate and identify the active protein, cell pellets of strain JH34071-1 were homogenized at ×30,000 psi after re-suspension in 20 mM Tris buffer, pH 8 with cOmplete™, EDTA-free protease inhibitor cocktail

(Roche, Indianapolis, IN, U.S.A.). The crude lysate was cleared by centrifugation and brought to 75% saturation with ammonium sulfate. The 75% ammonium sulfate solution was then centrifuged, and the supernatant was discarded. The pellet portion was suspended in 20 mM Tris pH 8.0 and then brought to 1.5 M ammonium sulfate with the addition of a 2 M ammonium sulfate, 20 mM Tris pH 8.0 solution. This solution was clarified and loaded onto a TSKgel™ Phenyl- 5PW column (Tosoh Bioscience, Tokyo, Japan) equilibrated in 20 mM Tris pH 8.0, 1.5 M ammonium sulfate. Insecticidal activity eluted with a gradient to 20 mM Tris, pH 8. Active fractions were pooled, concentrated on 10 kDa molecular weight cutoff centrifugal concentrators (Sartorius Stedim, Goettingen, Germany) and desalted into 20 mM piperazine pH 9.5 using a Sephadex G25 (GE Healthcare, Piscataway, NJ, U.S.A.) column. The desalted pool was loaded onto a Mono Q™ column (GE Healthcare) equilibrated in 20 mM piperazine, pH 9.5 and eluted with a gradient of 0 to 0.4 M NaCl. Active fractions were pooled and loaded onto a Superdex™ 200 column (GE Healthcare) equilibrated in phosphate buffered saline (PBS). SDS-PAGE analysis of fractions indicated that WCR activity coincided with a prominent band after staining with GelCode™ Blue Stain Reagent (Thermo Fisher Scientific). The protein band was excised, digested with trypsin and analyzed by nano-liquid chromatography/electrospray tandem mass spectrometry (nano-LC/ESLMS/MS) on a Thermo Q Exactive™ Orbitrap™ mass spectrometer (Thermo Fisher Scientific, 81 Wyman Street, Waltham, Mass. 02454) interfaced with an Eksigent™ NanoLC™ 1-D Plus nano-Ic system (AB Sciex™, Framingham, MA, U.S.A.). Protein identification was done by internal database searches using Mascot® (Matrix Science, London, UK). The candidate protein was designated originally as IPD090Aa (Mpf2Ba1). Cloning and recombinant expression confirmed the insecticidal activity of the Mpf2Ba1 polypeptide (accession #OP537915) against WCR. Note that for preparing plant expression constructs (see section below) the *mpf2ba1* gene sequence was modified to include an additional Ala immediately following the translation

initiation Met, thus increasing the amino acid reference numbers by one. Except as noted, all of the structure and biochemistry studies described below used this protein sequence.

Generation of Mpf2Ba1-1167

Simulated gastric fluid (SGF) is a common method for assessing the potential allergenicity of nonfood proteins[31]. It was observed that Mpf2Ba1 was stable when exposed to SGF (data not shown). An engineered SGF-susceptible variant, Mpf2Ba1-1167 (accession #OP575916), was generated by making five phenylalanine to tyrosine and four isoleucine to leucine mutations (Supplementary Table 1). Mpf2Ba1-1167 had increased susceptibility to SGF exposure while maintaining wild type potency against WCR (Fig. 1). Mpf2Ba1-1167 was used as a representative of Mpf2Ba1 for crystal structure determination.

Greenhouse efficacy of Mpf2Ba1-expressing plants

Greenhouse efficacy results for events generated from Mpf2Ba1 constructs are shown in Supplementary Fig. 1. Note that plant expression constructs utilized a modified *mpf2ba1* gene sequence that included a codon for an additional Ala immediately following the translation initiation Met. Events were generated using 13 different constructs designed to produce a range of Mpf2Ba1 accumulation. Constructs were introduced into *Agrobacterium tumefaciens* strain LBA4404, and those transformants were used to infect immature embryos of the maize inbred line PHR03 as described[32]. Efficacy for events derived from all 13 constructs was observed relative to negative control events (Empty) as measured by root protection from western corn rootworm. Root protection was measured according to the number of nodes of roots injured (CRWNIS = corn rootworm node injury score) using the method developed by Oleson, et al. (2005)[1]. The root injury score is measured from "0" to "3" with "0" indicating no visible root injury, "1" indicating 1 node of root damage, "2" indicating 2 nodes or root damage, and "3" indicating a maximum score of 3

nodes of root damage. Intermediate scores (e.g. 1.5) indicate additional fractions of nodes of damage (e.g. one and a half nodes injured).

Field efficacy of Mpf2Ba1-expressing plants

Field efficacy results for Mpf2Ba1 expressing plants are shown in Supplementary Fig. 1b. Events analyzed in this study were generated such that Mpf2Ba1 accumulated preferentially in the plastids of roots. In both Construct 1 and 2, Mpf2Ba1 transcript was driven by the SB-RCC3-PCOA118532A promoter (SEQ ID #12)[33] followed by the ZM-HPLV9 INTRON1 (SEQ ID #8)[34]. Transcript generation in both was terminated by the OS-T30 terminator (SEQ ID #31)[35]. The two constructs differed only in the transit peptide enabling plastid localization. In Construct 1, Mpf2Ba1 was targeted to plastids by fusing the Mpf2Ba1 transcript (minus the first Met codon) at the 5' end to nucleotides encoding:

`MCGSTPRTAKFIRAPHLGVKAWKMVAWPGSARWAKRLSMGPVPVYSAWNQAPMTASMARRPFLISLVR`.

In construct 2, the transit peptide was:

`MLIISSTPHLGVKAWKMVAWPGSARWAKRLSMGPVPVYSAWNQAPMTASMARRPFLISLVR`.

The experimental unit in the field was a single-row plot of corn 3 m in length and a row spacing of 76 cm. The experimental design was a randomized complete block with subsamples, planted at 8 locations, with treatments randomized within each of 3 replications. Treatments included 3 Mpf2Ba1 transformation events from Construct 2 (9 plots per location), 2 Mpf2Ba1 transformation events from Construct 1 (6 plots per location), 2 entries of the commercial event DAS-59122-7 as the positive control (6 plots per location), and 2 entries with no events for corn rootworm (CRW) protection as the negative control (6 plots per location). Treatments were evaluated from 4 locations that sustained sufficient root injury to the negative control (at least 0.75 nodes of injury). Additional experimental constructs not related to Mpf2Ba1 were included in the experiment but are not reported. The commercial event DAS-59122-7 expresses the

Gpp34Ab1/Tpp35Ab1 proteins from *Bacillus thuringiensis* strain PS149B1 that act together as a binary insecticidal protein to provide protection against corn rootworm CRW larvae[36]. All treatments were tested in a single hybrid with the same genetic background. A seed treatment containing the insecticide, thiamethoxam, at a rate of 0.25 mg a.i./kernel (Cruiser® 250; Syngenta Crop Protection, Inc., Greensboro, NC, USA) was applied to seeds in all treatments. This is the labeled rate for control of certain secondary insect pests of corn but does not control CRW.

The source of infested WCR eggs was a non-diapausing colony maintained by the Corteva Insectary Production Research group located in Johnston, IA. Root injury to the treatments was evaluated after the peak period of CRW larval feeding had occurred at each location. Roots were evaluated by digging a sub-sample of 3 roots per plot, washing the root systems clean of soil, and then visually assessing the amount of CRW larval injury (node-injury score) using the Iowa State 0-3 node-injury scale[1].

A linear mixed model was applied to model node-injury scores across locations. Data for node-injury score (Y_{ijmnks}) of location $(L)_i$, replication $(R)_j$, construct $(P)_m$, event $(E)_n$, plot $(K)_k$ and plant s, were modeled as a function of an overall mean μ, factors for location, location by replication, construct, event, location by construct, location by event, plot within each location $(K/L)_{ik}$ and a residual within each location $(\varepsilon/L)_{ijmnks}$. The model can be specified as:

$$Y_{ijmnks} = \mu + \underline{L_i} + \underline{(L \times R)_{ij}} + P_m + \underline{E_n} + \underline{(L \times P)_{im}} + \underline{(L \times E)_{in}} + \underline{(K/L)_{ik}} + \underline{(\varepsilon/L)_{ijmnks}}$$

where construct was treated as fixed effect, and all the other effects were treated as independent normally distributed random variables with means of zero. F-tests were used to assess significance for fixed effects. T-tests using standard errors from the model were conducted to compare treatment effects. A difference was considered statistically significant if the P-value of the difference was less than 0.05. All data analysis and comparisons were made in ASReml 3.0 (VSN International, Hemel Hempstead, UK, 2009).

Activity of Mpf2Ba1 against resistant strains of WCR

To demonstrate that Mpf2Ba1 works through a mechanism that is distinct compared to the WCR traits that are currently commercialized, we compared its activity against a laboratory susceptible population of WCR to its activity against a field-collected population from Readlyn, IA, an area where there is high use of commercialized traits (denoted as 'Readlyn'). The Readlyn population has been shown to have signs of resistance to both mCry3Aa and Gpp34Ab1/Tpp35Ab1 (see Supplementary Table 3).

Edman degradation sequencing

N-terminal cleavage was determined through Edman degradation sequencing, as described previously[37]. Untreated Mpf2Ba1 (2 mg) and Mpf2Ba1 processed with WCR gut fluid incubated at 37° C overnight (3 ml of 3 mg/ml Mpf2Ba1 mixture) were separated on a 4-12% Bis-Tris Gel and then transferred to a PVDF membrane using an IBlot® (ThermoFisher) semi-dry transfer system. The membrane was then stained with Sypro™ Ruby (ThermoFisher) to visualize protein bands and bands corresponding to untreated Mpf2Ba1, monomeric Mpf2Ba1, and oligomeric Mpf2Ba1 following gut fluid treatment were cut from the membrane and subjected to sequencing. Both the monomer and oligomeric bands revealed cleavage of 25 amino acids before Ala26 in Mpf2Ba1.

Liposome preparation

Liposomes were prepared from a mixture of 1-palmitoyl-2-oleoyl-glycero-3-phosphocholine (POPC 16:0-18:1; 10mg/mL Avanti Polar Lipids, Inc., Alabaster, Alabama) and 4% plant derived cholesterol (100 mg; Avanti Polar Lipids) in chloroform. The lipid mixture at 1 mg/mL was dried under a stream of nitrogen gas in a glass vial (Avanti Polar Lipids). The resulting thin film was rehydrated in 1x PBS buffer pH 7.4 (Gibco). The lipid suspension was vortexed, sonicated in an

ultrasonic water bath for 10 minutes at ~40˚C (Branson 1510 Ultrasonic Cleaner) and flash frozen in liquid nitrogen and thawed at least 3 times. The lipid mixture was then extruded through polycarbonate nanopore filters of 100 nm and 80 nm pore size (Avanti Polar Lipids) to generate large unilamellar liposomes of ~100 nm, using an Avanti Mini-Extruder with on a 40˚C heated plate (Grant Bio).

Negative stain EM

Carbon coated grids (EMS, 200 copper mesh) were cleaned and hydrophilized by glow discharge for 30 s (Pelco easiGlow, TedPella) at 25 mA before application of 4 µL of Mpf2Ba1 sample, diluted to 0.1 mg/mL. After 1 min waiting time, excess liquid was blotted away with filter paper (Whatman #1) and the sample was stained with 3 µL droplets of 2% uranyl formate (Genereon #24762-5). Images were acquired on a Tecnai F20 electron microscope (Thermo Fisher Scientific) with a DE20 camera (Direct Electron) at ×30k magnification (2.05 Å/pixel) and 0.75–1.5 µm underfocus.

Supplementary References

1. Oleson, J. D., Park, Y.-L., Nowatzki, T. M. & Tollefson, J. J. Node-injury scale to evaluate root injury by corn rootworms (coleoptera: Chrysomelidae). J Econ Entomol 98, 1–8 (2005).

2. Hu, X. T. & Owens, M. A. Multiplexed protein quantification in maize leaves by liquid chromatography coupled with tandem mass spectrometry: an alternative tool to immunoassays for target protein analysis in genetically engineered crops. J Agr Food Chem 59, 3551–3558 (2011).

3. Ashkenazy, H. *et al.* ConSurf 2016: an improved methodology to estimate and visualize evolutionary conservation in macromolecules. *Nucleic Acids Res* 44, W344–W350 (2016).

4. Xu, Q. *et al.* Structure of a membrane-attack complex/perforin (MACPF) family protein from the human gut symbiont *Bacteroides thetaiotaomicron*. *Acta Crystallogr Sect F Struct Biology Cryst Commun* 66, 1297–1305 (2010).

5. Ponting, C. P. Chlamydial homologues of the MACPF (MAC/perforin) domain. Curr Biol 9, R911–R913 (1999).

6. Goddard, T. D. *et al.* UCSF ChimeraX: Meeting modern challenges in visualization and analysis. *Protein Sci* 27, 14–25 (2018).

7. Meier, A. & Söding, J. Automatic prediction of protein 3D structures by probabilistic multi-template homology modelling. *Plos Comput Biol* 11, e1004343 (2015).

8. Waterhouse, A. M., Procter, J. B., Martin, D. M. A., Clamp, M. & Barton, G. J. Jalview Version 2 – a multiple sequence alignment editor and analysis workbench. Bioinformatics 25, 1189–1191 (2009).

9. Zaitseva, J. *et al.* Structure–function characterization of an insecticidal protein GNIP1Aa, a member of an MACPF and β-tripod families. *Proc National Acad Sci* 116, 201815547 (2019).

10. Rosado, C. J. *et al.* A common fold mediates vertebrate defense and bacterial attack. *Science* 317, 1548–1551 (2007).

11. Ni, T. et al. Structures of monomeric and oligomeric forms of the Toxoplasma gondii perforin-like protein 1. Sci Adv. 4, eaaq0762 (2018).

12. Law, R. H. P. et al. The structural basis for membrane binding and pore formation by lymphocyte perforin. Nature 468, 447–451 (2010).

13. Lovelace, L. L., Cooper, C. L., Sodetz, J. M. & Lebioda, L. Structure of human C8 protein provides mechanistic insight into membrane pore formation by complement. J Biol Chem 286, 17585–17592 (2011).

14. Spicer, B. A. et al. The first transmembrane region of complement component-9 acts as a brake on its self-assembly. Nat Commun 9, 3266 (2018).

15. Pang, S. S. *et al.* The cryo-EM structure of the acid activatable pore-forming immune effector Macrophage-expressed gene 1. *Nat Commun* 10, 4288 (2019).

16. Ni, T. *et al.* Structure and mechanism of bactericidal mammalian perforin-2, an ancient agent of innate immunity. *Sci Adv* 6, eaax8286 (2020).

17. Aleshin, A. E. et al. Structure of complement C6 suggests a mechanism for initiation and unidirectional, sequential assembly of membrane attack complex (MAC). J Biol Chem. 287, 10210–10222 (2012).

18. Lukoyanova, N. *et al.* Conformational changes during pore formation by the perforin-related protein pleurotolysin. *Plos Biol* 13, e1002049 (2015).

19. Chothia, C. & Murzin, A. G. New folds for all-β proteins. *Structure* 1, 217–222 (1993).

20. Boura, E. & Hurley, J. H. Structural basis for membrane targeting by the MVB12-associated β-prism domain of the human ESCRT-I MVB12 subunit. *Proc National Acad Sci* 109, 1901–1906 (2012).

21. Jumper, J. *et al.* Highly accurate protein structure prediction with AlphaFold. *Nature* 596, 583–589 (2021).

22. Pettersen, E. F. *et al.* UCSF Chimera – A visualization system for exploratory research and analysis. *J Comput Chem* 25, 1605–1612 (2004).

23. Joseph, A. P. *et al.* Refinement of atomic models in high resolution EM reconstructions using Flex-EM and local assessment. *Methods* 100, 42–49 (2016).

24. Farabella, I. *et al.* TEMPy: a Python library for assessment of three-dimensional electron microscopy density fits. *J Appl Crystallogr* 48, 1314–1323 (2015).

25. Pintilie, G. et al. Measurement of atom resolvability in cryo-EM maps with Q-scores. *Nat Methods* 17, 328–334 (2020).

26. Dun, Z., Mitchell, P. D. & Agosti, M. Estimating *Diabrotica virgifera virgifera* damage functions with field trial data: applying an unbalanced nested error component model. *J Appl Entomol* 134, 409–419 (2010).

27. Tinsley, N. A., Estes, R. E. & Gray, M. E. Validation of a nested error component model to estimate damage caused by corn rootworm larvae. *J Appl Entomol* 137, 161–169 (2013).

28. Schellenberger, U. *et al.* A selective insecticidal protein from *Pseudomonas* for controlling corn rootworms. *Science* 354, 634–637 (2016).

29. Wei, J. *et al.* A selective insecticidal protein from *Pseudomonas mosselii* for corn rootworm control. *Plant Biotechnol J* 16, 649–659 (2018).

30. Altschul, S. F., Gish, W., Miller, W., Myers, E. W. & Lipman, D. J. Basic local alignment search tool. *J Mol Biol* 215, 403–410 (1990).

31. Fu, T.-J., Abbott, U. R. & Hatzos, C. Digestibility of food allergens and nonallergenic proteins in simulated gastric fluid and simulated intestinal fluid-comparative study. *J Agr Food Chem* 50, 7154–7160 (2002).

32. Cho, M.-J. *et al.* Agrobacterium-mediated high-frequency transformation of an elite commercial maize (*Zea mays* L.) inbred line. *Plant Cell Rep* 33, 1767–1777 (2014).

33. Crow, A., Diehn, S., Sims, L. & Inventor; Pioneer Hi-Bred International, Inc, assignee. Plant regulatory elements and methods of use thereof. (2017).

34. Diehn, S. *et al.* Regulatory sequences for modulating transgene expression in plants. (2019).

35. Bhyri, P. *et al.* Plant terminator sequences. (2018).

36. Moellenbeck, D. J. *et al.* Insecticidal proteins from *Bacillus thuringiensis* protect corn from corn rootworms. *Nat Biotechnol* 19, 668–672 (2001).

37. Ochola J. B., *et al*. Identification of insect-selective and mammal-selective toxins from *Parabuthus leiosoma* venom. *Toxicon* 50:449-456 (2007).
