# Supplementary material for: Structural journey of an insecticidal protein against western corn rootworm
Source: Nat Commun. 2023 Jul 13;14:4171. doi: 10.1038/s41467-023-39891-7 (PMC10344926; doi:10.1038/s41467-023-39891-7)

Supplementary Movies Thumbnails & Legends

File Name: Supplementary Movie 1.

Description: detail of the β-prism rotation between Mpf2Ba1-1167 monomer to pre-pore transition. The single inter-domain H-bond between the bacterially conserved Glu137-Tyr460 breaks in the transition from monomer to pre-pore as a probable allosteric signal of membrane binding sent to the upper MACPF domain. The video supports data of Figure 2k.


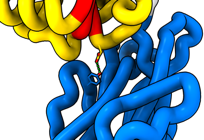


File Name: Supplementary Movie 2.

Description: Full journey into the conformational changes of Mpf2Ba1 from single monomer to pre-pore and pore subunit. The conformational changes of Mpf2Ba1 from crystal to pre-pore to pore are seen from different orientations.


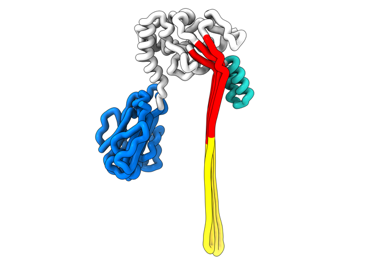


File name: Supplementary Movie 3.

Description: Full journey into the pre-pore to pore conversion of three Mpf2Ba1 single subunits. The conformational change from pre-pore to pore of three neighbouring Mpf2Ba1 subunits shows the rearrangement of TMHs and how the HTH motive is shifted laterally in order to reach contact with residues in TMH1 of the adjacent subunit, contributing to pore stabilization. The video supports data of Figure 3g and h.


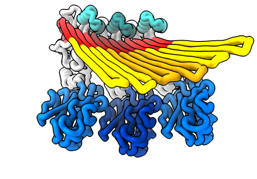

Supplement: Supplementary file 3 — Description of additional supplementary files [file 41467_2023_39891_MOESM3_ESM.docx]
